# Efficiency of voluntary auditory-speech memory in younger schoolchildren with different types of dysontogenesis

**DOI:** 10.1192/j.eurpsy.2022.1095

**Published:** 2022-09-01

**Authors:** M. Zvereva, N. Zvereva, E. Balakireva

**Affiliations:** 1 FSBSI MENTAL HEALTH RESEARCH CENTER, Clinical Psychology, Moscow, Russian Federation; 2 Mental Health Research Center, Child Psychiatry, Moscow, Russian Federation

**Keywords:** children with mental and psychic disorders, voluntary auditory-verbal memory

## Abstract

**Introduction:**

Assessing of voluntary auditory verbal memory, mediated or non-mediated by meaning context is an important component of impaired mental or psychic development estimation in children with different types of dysontogenesis.

**Objectives:**

Investigation of memory in chidren with mental disorders

**Methods:**

Participants: Children 9-12 years: ND - 25 normal development (14 boys), F70 - 31 with mild mental retardation (18 boys), F20.8 - 15 children with childhood type of schizophrenia (11 boys) and F21- 29 with schizotypal disorder (22 boys). Learning of 10 words, simple (SPA) and complex (CPA) paired associations. Assessing Parameter - auditory-speech memory efficiency in each of techniques. Mann-Whitney criterion.

**Results:**

There were significant differences in memory efficiency of 10 words between F21 and F70 (p≤0.01) and F70 and F20.8 (p≤0.05). Simple paired associations - no differences between all groups. Complex paired associations: F21 and F70 (p≤0.05), F21 and F20.8 (p≤0.01). ND - significant differences in memory efficiency of 10 words with all groups (F21 – p ≤0.01, F20.8 – p ≤0.05, F70 – p ≤0.01). SPA: significant differences with all groups (p≤ 0.01). CPA - significant differences words with all groups (F21 – p≤ 0.05, F20.8 – p≤ 0.01, F70 – p ≤0.01).

**Conclusions:**

Сommon features of working memory in children with diseased type of development:- improved memorization with introduction of strong semantic connections for all types of dysontogenesis. For complex connections in different groups we suppose different mechanisms of destroyed memory: F70 - low level of thinking, F20.8 - decreasing in mental activity or as a result of forming defect.

**Disclosure:**

No significant relationships.

